# Do some anxiety disorders belong to the prodrome of bipolar disorder? A clinical study combining retrospective and prospective methods to analyse the relationship between anxiety disorder and bipolar disorder from the perspective of biorhythms

**DOI:** 10.1186/s12888-017-1509-6

**Published:** 2017-10-24

**Authors:** Na Du, Ya-ling Zhou, Xu Zhang, Jing Guo, Xue-li Sun

**Affiliations:** 1 Department of Psychiatry, The Fourth People’s Hospital of Chengdu, Chengdu, 610031 China; 2 Department of Psychiatry, West China Hospital, Sichuan University, Chengdu, 610041 China

**Keywords:** Bipolar disorder, Anxiety disorders, Biorhythm

## Abstract

**Background:**

In clinical practice, some patients diagnosed with anxiety disorder (AD) may develop bipolar disorder (BD) many years later, and some cases of AD may be cured by the use of mood stabilizers. However, the relationship between AD and BD should be explored further.

**Method:**

To track how many cases of AD turned to BD and to discover the differences between them, we recruited 48 patients diagnosed with BD, who were assigned to the BD group for the retrospective analysis, and we also recruited 186 patients diagnosed with AD at enrolment; this latter group was asked to complete follow-up surveys conducted 3 months, 6 months, 12 months and 18 months after the primary stage of the study. We defined another two groups according to the usage of mood stabilizers, the rates of reduction in scores on the Hamilton Anxiety Scale and Yale-Brown Obsessive Compulsive Scale, and the changes in Clinical Global Impression scores at different follow-up times: the anxiety group and the atypical BD group (who used mood stabilizers to treat AD). All subjects also completed the NEO Five-Factor Inventory and supplied blood samples to be tested for several endocrine indices (TSH, T3, FT3, T4, FT4, ACTH,PTC) and inflammatory cytokines (IL-6, IL-8, IL-10, TNF-α, CRP) at enrolment.

**Results:**

In total, 14 subjects developed BD by the end of the study. One hundred eleven subjects were included in the anxiety group. Sixty-three subjects were assigned to the atypical BD group, and they had similar features to the 48 subjects in the BD group in terms of personality traits, abnormality rates of endocrine indices and levels of inflammatory cytokines. From the anxiety group to the atypical BD group and then the BD group, the age of first onset gradually decreased, while the frequency of onset and the score of suicidal ideation gradually increased. Furthermore, the atypical BD group showed markedly higher levels of TSH, IL-6, TNF-α and CRP than the other two groups.

**Conclusions:**

Some ADs with unique features might belong to the prodromal stage or the atypical presentation of BD, and recognizing these ADs early will economize many medical resources.

**Electronic supplementary material:**

The online version of this article (10.1186/s12888-017-1509-6) contains supplementary material, which is available to authorized users.

## Background

Bipolar disorder (BD), a type of mood disorder, generally refers to recurrent manic or hypomanic and depressive episodes that occur over a period of time. BD is a common mental illness with an early age of onset, high rates of prevalence, recurrence, morbidity and suicide, and a heavy disease burden [1]. When mild episodes are included, the incidence of BD in the population can reach 4.4% [2]; when atypical episodes (episodes that do not meet the diagnostic criteria of BD in the dimensions of time and severity) are also included, the incidence can reach 6.5% [3].

Biological processes that repeat approximately every 24 h and persist during the same period in the absence of external cues are defined as circadian rhythms [4]. The important physiological functions of the human body, including blood pressure and blood glucose, exhibit circadian fluctuations. Therefore, recent theories suggest that common diseases such as hypertension and diabetes may be caused by rhythm imbalance. In addition, BD, which is characterized by erratic changes in emotion, can also be understood as a dysrhythmia. Consequently, many studies have attempted to identify the pathogenesis of BD from the perspective of rhythm. For example, Westrich and Sprouse [5] found that some BD patients have a free-run pace of less than 24 h and that this shortening of the circadian period results in phase advances and usually precedes a hypomanic or manic episode. Moreover, Abreu [6] noted that BD symptoms, including mood, energy, sleep, appetite, and attention changes, all represent a change in rhythm, and some academics have suggested that mood stabilizers, the main drugs used to treat BD, work by influencing the biological activity of the circadian clock [7].

Most recent studies have focused on the nature of BD from only a narrow point of view, namely, circadian rhythm imbalance. However, from a broad perspective, the shift in circadian rhythms might also follow the pattern common to many other diseases, developing from a normal rhythm to an abnormal rhythm and then a pathological rhythm until decompensation occurs; these latter stages correspond to the prodrome, onset and complications of clinical disease. In the simple example of diabetes, which is characterized by dysrhythmia of blood glucose, impaired glucose tolerance is the prodromal phase. In this phase, the patient’s blood glucose shows abnormal rhythms that are still reversible. As the disease develops further, the patient is diagnosed with diabetes, indicating a pathological rhythm. As the disease continues to worsen, renal failure or other complications occur, and the patient enters the irreversible decompensation period. As a type of disease, BD might also follow the rules mentioned above, and the fluctuation of emotion, which is analogous in this case to blood glucose in diabetes, might follow the pattern of disease evolution. However, the current diagnostic criteria for BD list phenomenological symptoms for the onset period only and do not describe any features of the prodromal period or complications, which leads to a low rate of diagnosis, a poor response to drugs and an unsatisfactory prognosis. Hence, it is necessary to research the prodromal period of BD. Only through discovering symptoms of the prodromal period can we stop abnormal rhythms from developing into pathological rhythms that cannot be reversed. According to most of the recent studies in the literature, the prodromal symptoms of BD may include subthreshold manic symptoms, anxiety and other symptoms, and these symptoms are often atypical [8–12]. Zeschel et al. [13] performed a retrospective study of 42 BD patients and found that they all had certain prodromal symptoms, including instability and a change in life rhythms, before the onset of their manic or depressive episode. Some scholars have even noted that the duration of prodromal symptoms in BD might be 1.8 to 7.3 years. When that period is shorter, the rate of developing BD is higher and the symptoms are more severe [14]. However, out of several “prodromal” symptoms, anxiety symptoms have emerged as a priority. Patients with the chief complaint of anxiety often report poor efficacy in anxiolytic treatment and ultimately develop BD. It is difficult to make a correct diagnosis based on medical history unless the patient previously presented with mania and was hospitalized [15]. Therefore, it is necessary to study prodromal symptoms manifested as anxiety, which may have a profound impact on the treatment and prognosis of BD.

As a result, many scholars have begun to seek the relationship between BD and anxiety disorders. For example, the 2013 National Epidemiological Survey on Alcohol and Related Conditions (NESARC) found that in subjects with elation or irritability, generalized anxiety disorder can be a good predictor of later hypomania [16]. Another study found that subjects who did not have panic disorder before they were recruited had a higher rate of developing BD if they experienced a panic attack than if they did not have a panic attack [17]. Kessler et al. [18] found that anxiety disorders can predict the occurrence of BD even if no other variables are considered. Furthermore, Mesman et al. [19] used a prospective method to study patients with BD and their offspring and found an association between anxiety disorder and BD. They mentioned that anxiety disorder is likely to be the first pathological mental process in the development of BD. Similarly, Johnson et al. [20] reported that adolescents suffering from anxiety disorders are more likely to show significant clinical features of BD or manic symptoms in early adulthood than those without an anxiety disorder. Additionally, Faedda et al. [21] conducted a meta-analysis that showed that the early onsets of panic disorder, separation anxiety and generalized anxiety disorder were risk factors for the onset of typical BD within a few years. Rucklidge et al. [22] also argued that excessive anxiety and worry leading to social dysfunction showed high sensitivity and specificity for predicting the onset of BD.

These studies attempted to ascertain the relationship between anxiety and BD, but they failed to determine the special features of anxiety disorder that are most likely to develop into BD or to be an atypical expression of BD in terms of rhythm. They also failed to explain why and how anxiety becomes the prodromal period of BD. From the viewpoint of psychology, anxiety is a basic emotion that can be converted or transformed into any other emotion [23]. We hypothesize that when people experience stress, the common emotion of anxiety occurs, and with the continuation of stress, this emotion gradually evolves into pathological anxiety. From that moment on, the normal rhythm of emotion is disrupted, which indicates the beginning of the prodromal period of BD, although it is still manifested as anxiety disorder and lacks the typical symptoms of BD. If the stress persists, it may result in the recession of anxiety and the emergence of the depressive symptoms of BD. In such cases, the rhythm of emotion becomes pathological, a conversion that is difficult to reverse. If the stress continues, it may result in release (namely, disinhibition), which manifests as the manic symptoms of BD. Thus, for the group of patients who do not have depressive symptoms but first present with manic symptoms, the main mechanism of disease may be excessive self-protection caused by their personal qualities and other factors. This self-protection causes them to bypass the pain of depression and to move directly into the manic phase, which is accompanied by positive feelings about themselves. According to our hypothesis, there might be a period between anxiety and BD that we define as atypical BD. Atypical BD shows its symptoms in the form of anxiety disorders but simultaneously possesses special features of BD in psychological and neurobiological aspects. Because these special features can be either inherited or contracted, some patients with anxiety might stay in the stage of common anxiety disorder without developing BD, while others might progress to BD after many years. The most compelling evidence is that some anxiety patients may be cured by the use of mood stabilizers instead of anxiolytics. Hence, there is an urgent need to verify the existence of atypical BD and discover the differences that separate it from common anxiety disorders. If atypical BD exists as defined here, we believe that BD will also obey the developmental rule of rhythm and that anxiety disorders may occur in the first stage of its development.

Based on the hypothesis mentioned above, we chose patients diagnosed with anxiety disorders cured by the usage of either mood stabilizers or anxiolytics and included patients diagnosed with BD as the control group. We used a combination of retrospective and prospective methods to study the psychological and neurobiological features of all the subjects in terms of personality, endocrine indices and inflammatory cytokines for the following purposes: 1, to analyse the differences between anxiety patients cured by mood stabilizers and those cured by anxiolytics; 2, to determine whether the patients diagnosed with anxiety disorders but cured by mood stabilizers share some similarities with BD patients, which would further substantiate the existence of atypical BD; and 3, to explore and explain how anxiety disorders become the prodromal period of BD from the perspective of rhythm and to try to reveal why some common anxiety disorders do not develop into BD.

## Methods

### Subjects

#### Sample size and sources

The typical BD group comprised 48 patients diagnosed with BD. The simple anxiety group comprised 111 patients diagnosed with anxiety disorders, and the atypical BD group comprised 63 patients who also met the criteria for anxiety disorders but used mood stabilizers (the specific grouping rules are listed below). These patients were selected from the Mental Health Center of West China Hospital in Chengdu from May 2014 to September 2014 using the convenience sampling method.

#### Inclusion criteria

**Fig. 1:**
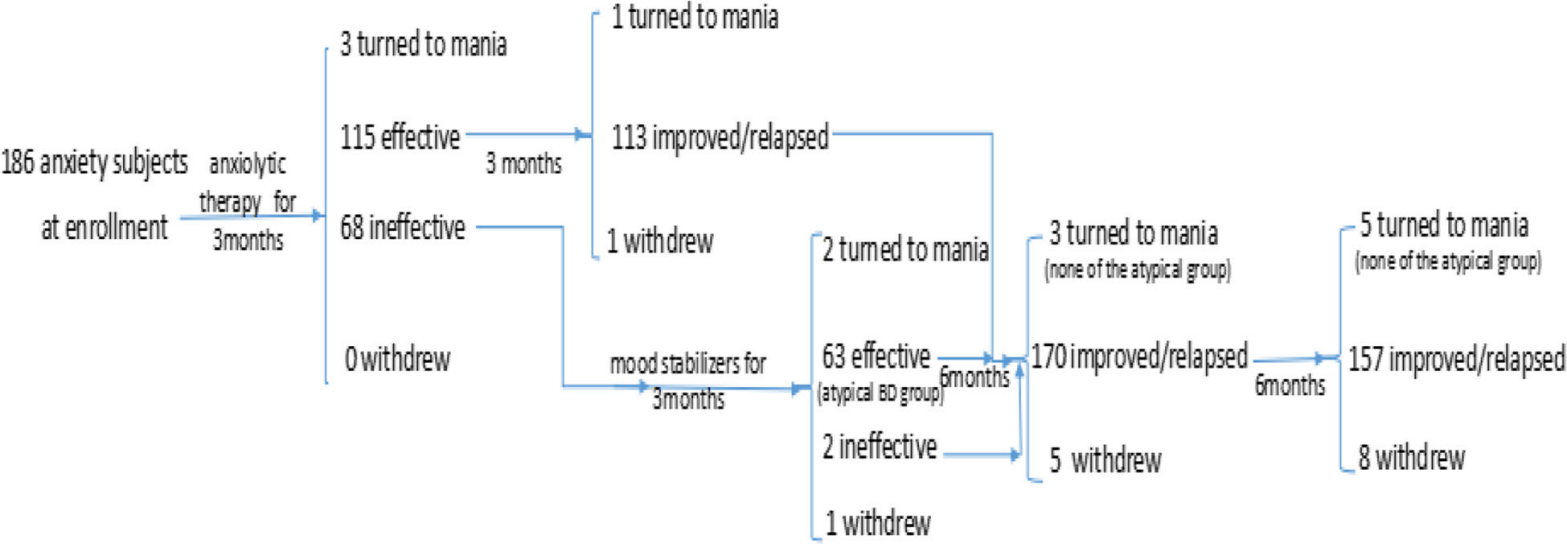
The outcome of subjects and the flow chart of follow-up

All the patients but those in the typical BD group needed to meet the International Classification of Diseases, Tenth Edition (ICD-10) criteria for anxiety disorders, including generalized anxiety disorder, panic disorder, obsessive-compulsive disorder (OCD), phobias, acute stress disorder, and post-traumatic stress disorder. These patients all received anxiolytic therapy during hospitalization and completed our 1.5-year follow-up study after they were discharged from West China Hospital. The first follow-up visit was conducted 3 months after the anxiolytic therapy, the second one was completed 6 months later, the third was completed 12 months later, and the last was completed 18 months later. The flow chart is shown in Fig. 1.

##### The simple anxiety group

All the subjects in the simple anxiety group met the criteria presented in inclusion criteria, and their treatment was assessed as effective at the first follow-up based on reductions in their clinical scale scores and their scores on the Clinical Global Impression (CGI) scale (i.e., reductions in both the Hamilton Anxiety Scale (HAMA) and the Yale-Brown Obsessive Compulsive Scale (YBOCS) ≥25% and an overall CGI ≤3), but patients who showed manic symptoms at subsequent follow-up visits were excluded.

##### The atypical BD group

All the subjects in the atypical BD group also met the criteria in inclusion criteria, and their treatment was assessed as ineffective or non-significant at the first follow-up based on their clinical scale and CGI scores. Furthermore, these patients were given mood stabilizers, such as valproate, lamotrigine or lithium carbonate, for treatment after anxiolytic therapy proved ineffective. The mood stabilizers were prescribed at the second follow-up visit, 3 months later. Patients who exhibited manic symptoms at subsequent follow-up visits were excluded.

##### The BD group

All the subjects in the BD group met the ICD-10 diagnostic criteria for bipolar disorder with anxiety disorders as their first-onset disease and were currently in the onset phase of BD rather than in remission.

##### Patients who progressed to mania during the follow-up process

Subjects who met the criteria in inclusion criteria and displayed manic symptoms during the follow-up period, as assessed by Young Mania Rating Scale (YMRS) scores and the duration of symptoms (i.e., YMRS scores ≥6 points and symptoms consistent with the ICD-10 criteria for a hypomanic/manic episode), were considered new BD patients.

#### Exclusion criteria

Anxiety patients with any of the following features were excluded:

1. suffering from non-anxiety mental disorders, such as BD, depression, or schizophrenia, or from severe physical illness (within the past 3 months) that might make the patient unable to complete the questionnaires;
2. unable to read or comprehend the questionnaires;
3. unwilling to participate or uncooperative.

Patients with typical BD who met any of the following exclusion criteria were not enrolled:

1. suffering from other mental disorders in addition to BD or from neurological diseases or severe physical illness (within the past 3 months) that might make them unable to complete the questionnaires;
2. Unable to read or comprehend the questionnaires;
3. unwilling to participate or uncooperative.

### Measures

#### Survey tools

##### The basic demographic questionnaire

The graphic questionnaire was designed by members of our research team to collect data regarding the participants’ demographic features and the clinical features of their diseases. (The intact version of this questionnaire can be found in the Additional file 1, and the specific items are listed in Tables 1, 2 and 3). The item on suicidal ideation was adapted from the 10th item of the Montgomery-Asberg Depression Rating Scale and was scored on a 6-point scale. The higher the score was, the more severe the symptom.

**Table 1:** Demographic characteristics

Characteristic		BD (*n* = 48)			Atypical (*n* = 63)			Anxiety (*n* = 111)			*χ* ^2^	F
		Mean ± SD	*n*	Percentage (%)	Mean ± SD	*n*	Percentage (%)	Mean ± SD	*n*	Percentage (%)		
Age (years)		33.06 ± 14.53			34.33 ± 15.55			37.38 ± 12.15				0.676
Sex	Male		15	31.3		18	28.6		39	35.1	0.276	
	Female		33	68.8		45	71.4		72	64.9		
Nationality	Han		45	93.8		60	95.2		105	94.6	0.039	
	Minority		3	6.3		3	4.8		6	5.4		
Birth place	SC		42	87.5		51	80.9		105	94.6	5.728	
	CQ		3	6.3		0	0		0	0		
	GZ		0	0		3	4.8		0	0		
	Others		3	6.3		9	14.3		6	5.4		
Residence	City		36	75		54	85.7		90	81.1	0.680	
	Village		12	25		9	14.3		21	18.9		
Education	Primary		0	0		0	0		9	8.1	5.267	
	Middle		12	25		15	23.8		24	21.6		
	High		24	50		27	42.9		33	29.7		
	Bachelor		12	25		21	33.3		45	40.5		
	Master		0	0		0	0		0	0		
Marriage	Unmarried		27	56.3		30	47.6		24	21.6	14.493**	
	Married		12	25		27	42.9		81	73		
	Divorced		6	12.5		6	9.5		6	5.4		
	Remarried		0	0		0	0		0	0		
	Widowed		0	6.3		0	0		0	0		
Work	None		24	50		24	38.1		30	27	3.926	
	Student		12	25		18	28.6		15	13.5		
	Worker		6	12.5		0	0		12	10.8		
	Teacher		3	6.3		3	4.8		9	8.1		
	Doctor		0	0		0	0		3	2.7		
	Civil servant		0	0		6	9.5		12	10.8		
	Staff		0	0		12	19		18	16.2		
	Others		3	6.3		0	0		12	10.8		

**P < 0.01

*Han* represents the Han nationality, *SC* represents Sichuan province, *CQ* represents Chongqing city, and *GZ* represents Guizhou province

**Table 2:** Clinical features of disease (categorical variables)

Variables		BD (*n* = 48)		Atypical (*n* = 63)		Anxiety (*n* = 111)		*χ* ^2^
		*n*	%	*n*	%	*n*	%	
Type of BD	Hypomania	0	0					
	Non psychotic mania	12	25					
	Psychotic mania	3	6.3					
	Mixed state	15	31.3					
	Remission	0	0					
	Mild depression	12	25					
	Non psychotic major depression	3	6.3					
	Psychotic major depression	3	6.3					
Type of anxiety	GAD			54	85.7	75	67.6	5.607
	OCD			6	9.5	27	24.3	
	Stress related disorders			3	4.8	0	0	
	Panic Disorder			0	0	9	8.1	
First onset	No	48	100	48	76.2	90	81.1	4.187
	Yes	0	0	15	23.8	21	18.9	
Type of first onset	GAD	36	75	57	90.5	78	70.3	2.090
	OCD	6	12.5	3	4.8	24	21.6	
	Stress related disorders	3	6.3	3	4.8	0	0	
	Panic Disorder	3	6.3	0	0	9	8.1	
Family history	No	42	87.5	51	81	99	89.2	0.796
	Yes	6	12.5	12	19	12	10.8	
Drug abuse	No	45	93.8	60	95.2	108	97.3	0.399
	Yes	3	6.3	3	4.8	3	2.7	
Other disease	No	45	93.8	51	81	87	78.4	1.867
	Yes	3	6.3	12	19	24	21.6

**Table 3:** Clinical features of disease (continuous variables)

Variables	BD (*n* = 48)	Atypical (*n* = 63)	Anxiety (*n* = 111)	F
	Mean ± SD	Mean ± SD	Mean ± SD	
Number of onsets	5.50 ± 2.53^a^	3.19 ± 1.81	3.29 ± 1.99^b^	7.351^**^
Age of first onset (years)	22.50 ± 10.69^c^	28.67 ± 13.76	30.62 ± 12.32	2.397
Months since diagnosis	64.87 ± 73.35	59.14 ± 60.24	54.84 ± 85.92	0.097
Frequency (number/year)	1.72 ± 2.87	1.15 ± 1.27	1.01 ± 1.54	0.820
Suicidal ideation	2.00 ± 2.07^d^	1.76 ± 2.09^e^	0.57 ± 1.42	4.996^**^

**P < 0.01

^a^The BD group was significantly higher than the atypical group (*P* = 0.000). ^b^The BD group was significantly higher than the anxiety group (*P* = 0.000). ^c^The BD group was significantly younger than the anxiety group (*P* = 0.016). ^d^The BD group was significantly higher than the anxiety group (*P* = 0.006). ^e^The atypical group was significantly higher than the anxiety group (*P* = 0.003)

##### Hamilton Anxiety scale (HAMA) [24]

The HAMA includes 14 items. Each item is answered on a 5-point scale. A total score lower than 6 indicates no anxiety. In addition, the scale can be divided into two factors: somatic and mental anxiety. The HAMA was used to assess all anxiety patients in our study and was administered by experienced psychiatrists at the time of enrolment and at the first and the second follow-up visits. We defined treatment as effective when the reduction rate was ≥25% and as markedly effective when the reduction rate was ≥50%.

##### Young Mania Rating Scale (YMRS) [24]

The YMRS comprises 11 items. The 1st, 2nd, 3rd, 4th, 7th, 10th, and 11th items are scored on a 4-point scale, while the remaining items are scored on an 8-point scale. A score of less than 6 indicates no mania. This scale was used to assess all the anxiety patients in our study and was administered by experienced psychiatrists at the time of enrolment and at the first, second, third and fourth follow-up visits.

##### Yale-Brown Obsessive-Compulsive Scale (YBOCS) [24]

The YBOCS consists of 10 items scored on a 5-point scale. The higher the score is, the more severe the patient’s condition. The scale was used to assess the anxiety patients with obsessive-compulsive symptoms in our study and was administered by experienced psychiatrists at the time of enrolment and at the first and second follow-up visits. We defined treatment as effective when the reduction rate was ≥25% and as markedly effective when the reduction rate was ≥50%.

##### Clinical Global Impression scale (CGI) [24]

The CGI consists of the Disease Severity and Overall Efficacy subscales, which assess the severity of the disease (7-point scale; the larger the number, the more serious the disease) and the efficacy of the treatment (7-point scale; the larger the number, the worse the efficacy), respectively. It was used to evaluate the efficacy of treatment and was administered by experienced psychiatrists at the time of enrolment and at the first and second follow-up visits.

##### NEO Five-Factor Inventory (NEO-FFI) [25]

The NEO-FFI consists of 60 items and includes a total of five subscales, each subscale comprising 12 items scored on a 5-point scale. The Chinese version of the inventory has been shown to have good reliability and validity [25]. The five personality features are as follows: (1) Neuroticism (N), which refers to the tendency to experience negative emotions. The typical low score is 20.4, while the typical high score is 38.8. (2) Extraversion (E), which refers to the level of active commitment to the outside world. The typical low score is 26, while the typical high score is 42. (3) Openness (O), which refers to the degree of imagination and curiosity. The typical low score is 32, while the typical high score is 47. (4) Agreeableness (A), which refers to the capacity for cooperation and maintaining harmony in society. The typical low score is 30, while the typical high score is 48. (5) Conscientiousness (C), which refers to organization, persistence, and motivation in terms of goal-oriented behaviour. It also reflects the degree of self-control and the ability to delay meeting needs. The typical low score is 36, while the typical high score is 44 [26].

#### Examination of endocrine axes

The hypothalamic-pituitary-thyroid (HPT) axis indices examined in this study included thyroid-stimulating hormone (TSH; normal level is 0.27–4.2 mU/L), triiodothyronine (T3; normal level is 1.3–3.1 nmol/L), thyroxine (T4; normal level is 62–164 nmol/L), free triiodothyronine (FT3; normal level is 3.60–7.50 pmol/L), and free thyroxine (FT4; normal level is 12.0–22.0 pmol/L). The hypothalamic-pituitary-adrenal (HPA) axis indices of interest included plasma total cortisol (PTC, normal level is 147.3–609.3 nmol/L) measured at 8:00 AM and adrenocorticotropic hormone (ACTH, normal level is 5.0–78 ng/L). Blood samples were drawn at the beginning of treatment and analysed by the laboratory of West China Hospital.

#### Examination of inflammatory cytokines

The inflammatory cytokines examined in this study included interleukin-6 (IL-6, normal level is 0.00–7.00 pg/mL), interleukin-8 (IL-8, normal level is 0–62 pg/mL), interleukin-10 (IL-10, normal level is 0.0–9.1 pg/mL), tumour necrosis factor-α (TNF-α, normal level is <8.1 pg/mL) and C-reactive protein (CRP, normal is <5 mg/L). Blood samples were drawn at the beginning of treatment and analysed by the laboratory of West China Hospital.

#### Data collection

This study used a combination of prospective and retrospective methods. In the retrospective part, we reviewed the clinical features of BD patients and compared them with those of the anxiety patients. In the prospective part, we tracked those anxiety patients to determine how their disease evolved. This innovative combination allowed us to discover the developmental rule of how anxiety turned to BD. Throughout the course of the study, no treatment plan was interfered with, and only the development of disease was recorded. The BD patients only needed to complete the basic demographic questionnaire, the NEO-FFI and a blood draw at the time of enrolment. All the subjects signed informed consent documents. The research was approved by the Institutional Review Board of West China Hospital. The study included 234 subjects, of whom 48 belonged to the BD group. At the time of enrolment, there were 186 anxiety patients; at the end of follow-up, there were 111 remaining patients who met the criteria for the simple anxiety group, 63 who met the criteria for the atypical BD group, and 14 who progressed to mania during the study. The withdrawal rates for the 3-month, 6-month, 12-month, and 18-month follow-up periods were 0, 1.1%, 2.9%, and 4.8%, respectively. (For the specific stages of grouping and the number of people lost to follow-up, see Fig. 1).

#### Statistical analysis

Descriptive statistics were computed for all variables. Differences between categorical variables were analysed with χ^2^ tests or a rank-sum test. The independent samples t-test was used to compare the HAMA, YMRS, and YBOCS scores of the atypical BD group and the simple anxiety group. ANOVA was used to analyse the differences among the three groups (i.e., the BD group, the atypical BD group, and the simple anxiety group) in NEO-FFI scores, endocrine indices, and inflammatory cytokines. Then, pairwise comparison was used to explore the differences between two groups at a time. (If the variance was homogeneous, Bonferroni correction was selected; if the variance was heterogeneous, Tamhane’s T2 was selected).

All statistical analyses were carried out using the Statistical Package for the Social Sciences (version 21.0). Statistical significance was set at *P* < 0.05.

## Results

### Basic conditions among the BD, atypical BD and simple anxiety groups

#### Demographic characteristics

The subjects’ demographic data are summarized in Table 1. The three groups were not significantly different (*P* > 0.05) in age, sex composition, nationality, place of birth, residence, education or work. Marital status among the three groups was significantly different (χ^2^ = 14.493, *P* < 0.01). Married was the most common marital status in the simple anxiety group, while unmarried was the most common status in the other groups.

#### Clinical features of disease among the three groups

Tables 2 and 3 show the clinical features of all participants. There was no significant difference (*P* > 0.05) between the atypical BD group and the simple anxiety group regarding the distribution of the type of anxiety. The clinical features were not significantly different among the three groups (*P* > 0.05) in whether the patients were at their first onset, the type of first onset, family history, history of drug abuse, history of other diseases, age of first onset, years since diagnosis or frequency of onset. The number of previous episodes and the suicidal ideation score were significantly different (*F* = 7.351, 4.996; *P* < 0.01) among the three groups.

The pairwise comparison results showed that the number of onsets in the BD group was significantly higher than those of the remaining two groups (*P* < 0.01); the age of first onset of the BD group was significantly younger than that of the simple anxiety group (*P* < 0.05); and the suicidal ideation score in the simple anxiety group was significantly lower than those in the other two groups (*P* < 0.05). (See Table 3 and notes).

#### Characteristics of symptoms between the atypical BD group and the simple anxiety group at enrolment

The differences in symptoms between the two groups are shown in Table 4. Due to the large number of items, only those with significant differences are listed here. The subjects in the atypical BD group received higher scores than those in the simple anxiety group on the items of sleep, irritability, disordered thought form and content, aggressive behaviour, and appearance. Their cognitive impairment and depression were more serious and their sensory symptoms, autonomic nervous system symptoms and anxiety level on performance were less serious than those of the simple anxiety group. The level of somatic anxiety of the simple anxiety group was more prominent than that of the atypical BD group. In addition, the subjects with obsessive-compulsive symptoms in the atypical BD group had more serious obsessive-compulsive symptoms than the corresponding ones from the simple anxiety group.

**Table 4:** YMRS, HAMA and YBOCS scores of the atypical and anxiety groups

Variables	Atypical (*n1* = 63, *n*2 = 6) Mean ± SD	Anxiety (*n1* = 111, *n*2 = 27) Mean ± SD	t
YMRS-4 (sleep)	0.67 ± 0.90	0.03 ± 0.16	4.569^***^
YMRS-5 (irritability)	0.86 ± 0.95	0.14 ± 0.48	4.698^***^
YMRS-7 (thought form)	0.05 ± 0.22	0	2.340^*^
YMRS-8 (thought content)	0.14 ± 0.35	0	4.214^***^
YMRS-9 (aggressive behaviour)	0.05 ± 0.22	0	2.340^*^
YMRS-10 (appearance)	0.05 ± 0.22	0	2.340^*^
Total score of YMRS	1.81 ± 1.94	0.16 ± 0.49	5.433^***^
HAMA-5 (cognitive function)	1.90 ± 0.53	1.54 ± 0.83	2.646^**^
HAMA-6 (depressed mood)	2.05 ± 1.06	1.46 ± 0.86	3.534^**^
HAMA-8 (sensory symptoms)	0.67 ± 1.00	1.35 ± 1.24	−3.200^**^
HAMA-13 (ANS symptoms)	1.14 ± 0.65	1.46 ± 0.89	−2.099^*^
HAMA-14 (performance)	1.57 ± 0.67	1.97 ± 0.49	−3.546^**^
Somatic anxiety factor	4.14 ± 3.82	5.76 ± 3.46	−2.503^*^
Total score of YBOCS	22.50 ± 4.95	19.22 ± 2.95	2.052^*^

*P < 0.05, **P < 0.01, ***P < 0.001

*ANS* represents the autonomic nervous system, *n1* represents the number of subjects who completed the YMRS and the HAMA, *n2* represents the number of subjects who completed the YBOCS

### Comparing efficacy of treatment between the atypical BD and simple anxiety groups

The HAMA and YBOCS in the different follow-up periods are shown in Table 5. No significant difference (*t* = 1.247; *P* > 0.05) in total HAMA score was found between the two groups at enrolment, but a difference did exist in total YBOCS score (*t* = 2.052; *P* < 0.05); specifically, the atypical BD group scored significantly higher than the simple anxiety group. There were significant differences between the two groups in total HAMA and YBOCS scores of at the first follow-up (*t* = 2.328, 3.214; *P* < 0.05). The total HAMA and YBOCS scores of the atypical BD group at the second follow-up had no significant difference compared with the corresponding scores of the simple anxiety group at the first follow-up (*t* = −0.912, 1.385; *P* > 0.05). This means that at the second follow-up, the severity of symptoms of the atypical BD group was similar to that of the simple anxiety group. The final reduction rates of HAMA and YBOCS scores between the two groups were not significantly different (χ^2^ = 0.659, 0.852; *P* > 0.05). Thus, it was evident that all the symptoms of the subjects in both groups were improved after appropriate treatment. In other words, the treatment was effective.

**Table 5:** The reduction rates of HAMA and YBOCS scores in the atypical and anxiety groups

Variables	Anxiety (*n1* = 111, *n*2 = 27)			Atypical (*n1* = 63, *n*2 = 6)					*χ* ^2^
	Enrolment	3 months later	Reduction rate^a^	Enrolment	3 months later	6 months later	Reduction rate^b^	Reduction rate^c^	
Total HAMA score	16.92 ± 3.26	5.58 ± 1.46**	67.02	16.13 ± 3.98	14.82 ± 3.15	5.34 ± 1.21	8.12	66.90	0.659
Total YBOCS score	19.22 ± 2.95	8.36 ± 2.69**	56.50	22.50 ± 4.95	20.69 ± 4.35	9.58 ± 2.87	8.04	57.42	0.852

**P < 0.01

*n1* represents the number of subjects who completed the HAMA, *n2* represents the number of subjects who completed the YBOCS, ^a^represents the reduction rate between enrolment and 3 months later in the anxiety group; ^b^represents the reduction rate between enrolment and 3 months later in the atypical group; ^c^represents the reduction rate between enrolment and 6 months later in the atypical group; the value of *χ* ^2^represents the comparison between reduction rate a and reduction rate c

The CGI results over different follow-up periods are shown in Table 6. The distributions of the disease severity and overall efficacy of the simple anxiety group were significantly different between the time of enrolment and the time of first follow-up (Z = 2.583, 3.689; *P* < 0.01). The distribution of the disease severity and overall efficacy of the atypical BD group were not significantly different between the time of enrolment and the time of first follow-up (Z = 0.674, 0.968; *P* > 0.05), while there was a significant difference between the time of enrolment and the time of the second follow-up (Z = 2.435, 2.874; *P* < 0.01). Thus, it could be proved that all the symptoms of the subjects from both groups were improved after appropriate treatment. In other words, the treatment was effective.

**Table 6:** The changes in CGI score in the atypical group and the anxiety group

Variables	Anxiety (*n* = 111)			Atypical (*n* = 63)				
	Enrolment *n *(%)	3 months later *n *(%)	Z_1_	Enrolment *n *(%)	3 months later *n *(%)	6 months later *n *(%)	Z_2_	Z_3_
Disease severity			2.583^**^				0.674	2.435^**^
No assessment	0	0		0	0	0		
Normal	0	5 (4.5)		0	0	4 (6.3)		
Borderline	0	35 (31.5)		0	0	23 (36.5)		
Mild	10 (9.0)	55 (49.5)		9 (14.3)	12 (19.0)	27 (42.9)		
Moderate	36 (32.4)	16 (14.5)		22 (34.9)	25 (39.7)	9 (14.3)		
Obvious	45 (40.5)	0		24 (38.1)	19 (30.2)	0		
Severe	12 (10.8)	0		7 (11.1)	6 (9.5)	0		
Most severe	8 (7.3)	0		1 (1.6)	1 (1.6)	0		
Overall efficacy			3.689^***^					
No assessment	111	0		63	0	0	0.968	2.874^**^
Obviously improved	0	64 (57.7)		0	0	41 (65.1)		
Improved	0	39 (35.1)		0	0	16 (25.4)		
Slightly improved	0	8 (7.2)		0	0	6 (9.5)		
No change	0	0		0	61 (96.8)	0		
Slightly deteriorated	0	0		0	2 (3.2)	0		
Deteriorative	0	0		0	0	0		
Obviously deteriorated	0	0		0	0	0		

**P < 0.01, ***P < 0.001

*Z1* represents the comparison between enrolment and 3 months later in the anxiety group, *Z2* represents the comparison between enrolment and 3 months later in the atypical group, *Z3* represents the comparison between enrolment and 6 months later in the atypical group

### Differences in big five personality traits among the BD, atypical BD and simple anxiety groups

#### Differences in big five personality traits among the three groups

The extraversion, openness, agreeableness and conscientiousness scores were all significantly different among the three groups (*F* = 6.863, 5.545, 17.593, 11.472; *P* < 0.05), while no significant difference was found on the neuroticism score (*P* > 0.05).

#### Pairwise comparisons among the three groups

The pairwise comparison results showed the following: on the dimension of extraversion, the simple anxiety group had higher scores than the atypical BD group or the BD group, and the difference was statistically significant (*P* < 0.05); on the dimension of openness, the simple anxiety group had lower scores than the atypical BD group or the BD group, and the difference was statistically significant (*P* < 0.05); on the dimension of agreeableness, the simple anxiety group had higher scores than the atypical BD group or the BD group, and the difference was statistically significant (*P* < 0.05); and on the dimension of conscientiousness, the simple anxiety group had higher scores than the atypical BD group or the BD group, and the difference was statistically significant (*P* < 0.05) (see Table 7 and notes).

**Table 7:** Differences in Big Five personality traits among the BD, atypical BD and simple anxiety groups

Variables	Typical points low/high	BD (*n* = 48) Mean ± SD	Atypical (*n* = 63) Mean ± SD	Anxiety (*n* = 111) Mean ± SD	F
Neuroticism	20.4/38.8	43.63 ± 6.50	43.81 ± 5.74	42.59 ± 6.78	0.291
Extraversion	26/42	31.81 ± 6.36	32.95 ± 4.79^a^	36.81 ± 4.74^b^	6.863^**^
Openness	32/47	42.31 ± 6.22	41.24 ± 6.13^c^	37.41 ± 5.06^d^	5.545^**^
Agreeableness	30/48	34.44 ± 2.89	33.76 ± 3.83^e^	39.51 ± 4.41^f^	17.593^***^
Conscientiousness	36/44	34.38 ± 4.05	35.67 ± 3.73^g^	39.32 ± 3.87^h^	11.472^***^

**P < 0.01, ***P < 0.001

^a^The anxiety group was significantly higher than the atypical group (*P* = 0.000). ^b^The anxiety group was significantly higher than the BD group (*P* = 0.000). ^c^The anxiety group was significantly lower than the atypical group (*P* = 0.000). ^d^The anxiety group was significantly lower than the BD group (*P* = 0.003). ^e^The anxiety group was significantly higher than the atypical group (*P* = 0.000). ^f^The anxiety group was significantly higher than the BD group (*P* = 0.000). ^g^The anxiety group was significantly higher than the atypical group (*P* = 0.000). ^h^The anxiety group was significantly higher than the BD group (*P* = 0.000)

### Differences in endocrine indices among the BD, atypical BD and simple anxiety groups

#### Abnormality rates of endocrine indices among the three groups

The abnormality rates of the three groups are shown in Table 8. There was no significant difference (*P* > 0.05) in the abnormality rate of any index of the HPT axis or the HPA axis between the BD group and the atypical BD group. The abnormality rates of TSH, FT3, FT4 and ACTH in both the BD group and the atypical BD group were all significantly higher than those in the simple anxiety group (*P* < 0.01).

**Table 8:** Abnormality rates of endocrine indices among the BD, atypical BD and simple anxiety groups

Variables		BD (*n* = 48)	Atypical (*n* = 63)	Anxiety (*n* = 111)	*χ* ^2a^	*χ* ^2b^	*χ* ^2c^
		*n *(%)	*n *(%)	*n* (%)			
HPT	TSH	12 (25.0)	18 (28.6)	3 (2.7)	0.074	9.412^**^	20.227^***^
	T3	0 (0)	0 (0)	0 (0)	–	–	–
	FT3	3 (6.3)	5 (7.9)	0 (0)	2.671	6.993^**^	7.012^**^
	T4	0 (0)	0 (0)	0 (0)	–	–	–
	FT4	3 (6.3)	5 (7.9)	0 (0)	0.158	6.993^**^	7.012^**^
HPA	ACTH	6 (12.5)	6 (9.5)	0 (0)	0.111	7.186^**^	7.132^**^
	PTC	6 (12.5)	21 (33.3)	30 (27.0)	2.517	1.566	0.591

**P < 0.01, ***P < 0.001

^a^represents the comparison between the BD group and the atypical group; ^b^represents the comparison between the BD group and the anxiety group; ^c^represents the comparison between the atypical group and the anxiety group

#### Differences in endocrine indices among the three groups

The endocrine indices of the three groups are shown in Table 9. All seven indices of the HPT axis and HPA axis among the three groups were significantly different (*F* = 11.321, 7.638, 4.995, 6.820, 4.937, 5.808, 13.325; *P* < 0.05).

**Table 9:** Differences in endocrine indices among the BD, atypical BD and simple anxiety groups

Variables		BD (*n* = 48) Mean ± SD	Atypical (*n* = 63) Mean ± SD	Anxiety (*n* = 111) Mean ± SD	F
HPT	TSH	2.80 ± 1.62	4.44 ± 2.87^a^	2.14 ± 0.73^b^	11.321^***^
	T3	2.24 ± 0.42^c^	2.36 ± 0.36	1.98 ± 0.36^d^	7.638^**^
	FT3	4.45 ± 0.51	4.98 ± 0.69^e^	4.94 ± 0.49 ^f^	4.995^**^
	T4	91.22 ± 19.59	92.92 ± 18.43^g^	79.25 ± 10.12^h^	6.820^**^
	FT4	17.26 ± 3.30	17.07 ± 2.89^i^	15.32 ± 1.82 ^j^	4.937^*^
HPA	ACTH	21.96 ± 11.06	37.06 ± 27.58^k^	41.80 ± 16.64^l^	5.808^**^
	PTC	341.68 ± 151.71	544.82 ± 152.71^m^	540.25 ± 121.16^n^	13.325^***^

*P < 0.05, **P < 0.01, ***P < 0.001

^a^The atypical group was significantly higher than the BD group (*P* = 0.002). ^b^The atypical group was significantly higher than the anxiety group (*P* = 0.000)

^c^The BD group was significantly higher than the anxiety group (*P* = 0.026). ^d^The atypical group was significantly higher than the anxiety group (*P* = 0.000)

^e^The atypical group was significantly higher than the BD group (*P* = 0.004). ^f^The anxiety group was significantly higher than the BD group (*P* = 0.004)

^g^The atypical group was significantly higher than the anxiety group (*P* = 0.000). ^h^The BD group was significantly higher than the anxiety group (*P* = 0.003)

^i^The atypical group was significantly higher than the anxiety group (*P* = 0.000). ^j^The BD group was significantly higher than the anxiety group (*P* = 0.005)

^k^The atypical group was significantly higher than the BD group (*P* = 0.026). ^l^The anxiety group was significantly higher than the BD group (*P* = 0.001)

^m^The atypical group was significantly higher than the BD group (*P* = 0.000). ^n^The anxiety group was significantly higher than the BD group (*P* = 0.000)

#### Pairwise comparison among the three groups

The pairwise comparison results showed the following: the level of TSH was higher in the atypical BD group than in the BD group or the simple anxiety group, and the difference was statistically significant (*P* < 0.05); the level of T3 was lower in the simple anxiety group than in the atypical BD group or the BD group, and the difference was statistically significant (*P* < 0.05); the level of FT3 was lower in the BD group than in the atypical BD group or the simple anxiety group, and the difference was statistically significant (*P* < 0.05); the level of T4 was lower in the simple anxiety group than in the atypical BD group or the BD group, and the difference was statistically significant (*P* < 0.05); the level of FT4 was lower in the simple anxiety group than in the atypical BD group or the BD group, and the difference was statistically significant (*P* < 0.05); the level of ACTH was lower in the BD group than in the atypical BD group or the simple anxiety group, and the difference was statistically significant (*P* < 0.05); and the level of PTC was lower in the BD group than in the atypical BD group or the simple anxiety group, and the difference was statistically significant (*P* < 0.05) (see Table 9 and notes).

### Differences in inflammatory cytokines among the BD, atypical BD and simple anxiety groups

#### Abnormality rates of inflammatory cytokines among the three groups

The abnormality rates of the three groups are shown in Table 10. There were no significant differences (*P* > 0.05) in the abnormality rates of inflammatory cytokines between the BD group and the atypical BD group. The abnormality rates of IL-6, IL-8, TNF-α and CRP in both the BD group and the atypical BD group were all significantly higher than those in the simple anxiety group (*P* < 0.05).

**Table 10:** Abnormality rates of inflammatory cytokines among the BD, atypical BD and simple anxiety groups

Variables	BD (*n* = 48)	Atypical (*n* = 63)	Anxiety (*n* = 111)	*χ* ^2a^	*χ* ^2b^	*χ* ^2c^
	*n* (%)	*n* (%)	*n* (%)			
IL-6	9(18.8)	9(14.3)	3(2.7)	0.176	4.832^*^	7.384^**^
IL-8	4(8.3)	6(9.5)	0(0)	0.016	7.074^**^	7.132^**^
IL-10	0(0)	0(0)	0(0)	–	–	–
TNF	21(43.8)	30(47.6)	15(13.5)	0.070	8.927^**^	18.203^***^
CRP	3(6.3)	3(4.8)	0(0)	0.052	6.993^**^	5.356^*^

*P < 0.05, **P < 0.01, ***P < 0.001

^a^represents the comparison between the BD group and the atypical group; ^b^represents the comparison between the BD group and the anxiety group; ^c^represents the comparison between the atypical group and the anxiety group

#### Differences in inflammatory cytokine levels among the three groups

The inflammatory cytokine levels of the three groups are shown in Table 11. There were significant differences in the levels of IL-6, IL-10, TNF-α and CRP among the three groups (*F* = 9.022, 12.193, 8.632, 8.708; *P* < 0.001).

**Table 11:** Differences in inflammatory cytokine levels among the BD, atypical BD and simple anxiety groups

Variables	BD (*n* = 48) Mean ± SD	Atypical (*n* = 63) Mean ± SD	Anxiety (*n* = 111) Mean ± SD	F
IL-6	3.52 ± 3.54	6.42 ± 4.19^a^	2.53 ± 2.72^b^	9.022^***^
IL-8	27.92 ± 55.31	22.86 ± 40.00	24.76 ± 78.82	0.025
IL-10	4.66 ± 0.55	3.49 ± 0.86^c^	4.43 ± 0.86^d^	12.193^***^
TNF	7.69 ± 3.13	10.89 ± 7.76^e^	5.67 ± 2.08^f^	8.632^***^
CRP	2.84 ± 3.38	4.44 ± 3.83^g^	2.62 ± 2.85^h^	8.708^***^

***P < 0.001

^a^The atypical group was significantly higher than the BD group (*P* = 0.007). ^b^The atypical group was significantly higher than the anxiety group (*P* = 0.000). ^c^The atypical group was significantly lower than the BD group (*P* = 0.000). ^d^The atypical group was significantly lower than the anxiety group (*P* = 0.000). ^e^The atypical group was significantly higher than the BD group (*P* = 0.035). ^f^The atypical group was significantly higher than the anxiety group (*P* = 0.000). ^g^The atypical group was significantly higher than the BD group (*P* = 0.042). ^h^The atypical group was significantly higher than the anxiety group (*P* = 0.000)

#### Pairwise comparisons among the three groups

The pairwise comparison results showed the following: the level of IL-6 was higher in the atypical BD group than in the BD group or the simple anxiety group, and the difference was statistically significant (*P* < 0.05); the level of IL-10 was lower in the atypical BD group than in the BD group or the simple anxiety group, and the difference was statistically significant (*P* < 0.05); the level of TNF-α was higher in the atypical BD group than in the BD group or the simple anxiety group, and the difference was statistically significant (*P* < 0.05); and the level of CRP was significantly higher in the atypical BD group than in the BD group or the simple anxiety group, and the difference was statistically significant (*P* < 0.05) (see Table 11 and notes).

### The rate of progression to mania

Through the entire follow-up, a total of 14 people developed mania (the number of patients developing mania in different periods is listed in Fig. 1), representing 7.53% of the number of subjects in the prospective study. Their demographic data were not significantly different from those of the remaining anxiety patients (*P* > 0.05).

## Discussion

 This is the first study to explore the links and differences between anxiety disorders and BD from the perspective of rhythm to determine which type of anxiety disorder may be associated with the atypical form of BD and to compare this anxiety disorder with common anxiety disorders to identify their neurobiological and psychological differences. These investigations were an attempt to show the existence of a prodromal period of BD, as well as to provide some basis for the argument that BD may also abide by the law of disease development and that the essence of BD may be the dysrhythmia of anxious mood.

### The clinical features of the three groups

The results of the present study show that the number of onsets in typical BD patients was significantly higher than those in the other groups, and their age at onset was significantly lower than that of the patients in the simple anxiety group but not significantly different from that of the patients in the atypical BD group. Furthermore, their suicidal ideation scores did not differ significantly from those of the atypical BD group but were significantly higher than those of the simple anxiety group, that is, the patients in the simple anxiety group had a relatively low suicidal ideation score. These findings suggest that the features of the atypical BD group were similar to those of the BD group but different from those of the simple anxiety group. The trend of the data shows that from the simple anxiety group to the atypical BD group and then the BD group, the age of onset decreased, while the frequency of onset gradually increased. The suicidal ideation score also showed a gradual increasing trend. It was possible to infer that the patients in the atypical BD group were in a transition period of BD, characterized by a shift from an abnormal rhythm to a pathological rhythm. With anxiety beginning first, some patients sharing the characteristics of BD (such as severe suicidal ideation or young age of onset) may have continued to deteriorate when not treated with mood stabilizers. These patients may have been at the stage of abnormal rhythm at this time, namely, the stage of atypical BD — the prodromal period of BD. Because their rhythm had not yet become pathological, their symptoms were atypical and difficult to recognize. In addition, according to previous reports, suicide is the leading cause of death in mood disorder patients [1], and every 10 patients with BD attempt suicide before their first manic episode [27]. Therefore, the existence and severity of suicidal ideation may be the turning point for distinguishing whether anxiety disorders are indicative of BD.

The YMRS, HAMA and YBOCS results at enrolment showed that the patients in the atypical BD group had more prominent symptoms related to sleep, irritability, the form and content of thought disorder, aggressive behaviour and appearance compared with the patients in the simple anxiety group. Furthermore, their cognitive dysfunction (mainly in terms of inattention and poor memory) and depressed mood were more serious, while their sensory symptoms, autonomic neurological symptoms and anxious behaviour when talking were less serious. However, the somatic anxiety of the patients in the simple anxiety group was more prominent. These results demonstrate that the clinical features of disease in the atypical BD group and the simple anxiety group differ somewhat. Studies have shown that before typical BD symptoms appear, the patient will present with a series of atypical symptoms and disruptive changes in biorhythms, such as decreased sleep requirements, an inability to concentrate, irritability, decreased energy, fatigue, and anhedonia [13]. Therefore, in our study, the symptoms of the atypical BD group that differed from those of the patients in the simple anxiety group may confirm the presence of prodromal symptoms of BD. Recent epidemiological and clinical studies show a strong correlation between OCD and mood disorders [28]. Some scholars have even suggested that the subset of OCD characterized by an episodic course may itself represent an atypical form of BD rather than a simple comorbidity [29]. Therefore, this study’s finding that the YBOCS score of the atypical BD group was significantly higher than that of the simple anxiety group could further support the existence of atypical BD.

### Big five personality trait differences among the three groups

The results of this study indicate that there was no significant difference in any factor of the NEO-FFI between the typical BD group and the atypical BD group, while all factors of these two groups differed significantly from those of the simple anxiety group. This finding shows that the assessment of personality characteristics can distinguish BD from normal anxiety disorders. This link to personality may be an inherent reason why some patients with anxiety disorders stay at the abnormal rhythm stage rather than progress to a pathological rhythm, namely, BD.

The results of the NEO-FFI indicate that the three groups did not differ significantly in the neuroticism factor, which illustrates that the patients in the three groups were more inclined to experience negative emotions and often had a poor ability to regulate their emotions. Chen et al. [30] also found that patients with anxiety disorders had obvious neurotic personality tendencies. Barnett et al. [31] reported that compared with the general population, BD patients also tended to present a high degree of nervousness, as measured with the NEO-FFI. The simple anxiety group’s score on the extraversion factor was significantly higher than that of the remaining two groups, suggesting that patients with BD are often quieter and less interested in the outside world than patients with simple anxiety. This finding is consistent with the results of Barnett et al. [31]. On the openness factor, the scores of the typical BD group and the atypical BD group were significantly higher than those of the simple anxiety group, which shows that patients with BD are often imaginative and creative. One possible reason is that BD patients have a more open attitude and more easily accept new things, especially during manic episodes [32]. Nowakowska et al. [33] used the NEO-FFI to assess BD patients and found that they scored higher than the control group on the openness factor. Galvez et al. [34] also discovered that BD patients have a considerable degree of creativity, realism and compliance. Conversely, patients with anxiety disorders tend to be pragmatic, traditional and conservative, which can be confirmed by their highly sensitive, cautious and alert personalities. On the agreeableness factor, the scores of the typical BD group and the atypical BD group were significantly lower than those of the simple anxiety group, indicating that patients with BD are generally reluctant to care for others and that they present certain problems in terms of social cooperation and harmony. Gruber et al. [35] also revealed that patients with BD tend to show less compassion. On the conscientiousness factor, the scores of the typical and atypical BD groups were significantly lower than those of the simple anxiety group, indicating that the behaviour of patients with BD is not standardized and that they are careless and have low work efficiency; these findings are reflected by the clinical characteristics of BD patients. This result is also consistent with those of Barnett et al. [31] and Nowakowska et al. [33]. Overall, anxiety patients with the personality traits of poor stability, poor agreeableness, and low conscientiousness may actually be presenting with the prodromal stage of BD.

### Differences in endocrine indicators among the three groups

The results of our study suggest that the differences in the abnormality rates of the HPT and HPA axis indicators between the typical BD group and the atypical BD group were not significant and that these values were all significantly higher in the typical and atypical BD groups than in the simple anxiety group. This lack of separation between typical and atypical BD demonstrates that the patients in the atypical BD group are essentially similar to those of the BD group from an endocrinological perspective. Although most patients with BD have no apparent thyroid disease, subtle changes in thyroid function are often found during examinations [36], and related studies indicate that HPT axis dysfunction may represent a potential phenotype of BD [37]. The HPA axis is the body’s main neuroendocrine system for coping with stress and adjusting emotions and mood [38]. It is clear that impairment in the function of the HPA axis is closely correlated with mood disorders [39], which explains why the BD patients and atypical BD patients in our study had higher abnormality rates of HPA axis values. Remlinger-Molenda et al. [40] also speculated that HPA axis dysfunction may be a characteristic of BD patients.

The results of the pairwise comparison showed that in terms of TSH, the atypical BD group had significantly higher values than the other two groups, which may explain why some patients in the prodromal stage of BD do not exhibit typical characteristics of BD. Those patients might have their own specific characteristics that could cause their anxiety to develop closer to BD. Regarding T3, the simple anxiety group had significantly lower values than the other two groups. This difference may be due to the activation of their HPA axis, which may suppress the relatively inactive thyroid hormone in the peripheral tissue from transforming into T3, which prompts the body to store more energy in response to stress [41]. This result further confirmed that anxiety might be the basic emotion involved in the development of BD. Regarding FT3, the typical BD group had significantly lower values than the other two groups. Mendonca et al. [42] also found that patients with BD had decreased FT3 and that the degree of decrease had a negative correlation with the severity of the disease. This result tells us only that atypical BD usually shares some features with common anxiety disorders, making them difficult to recognize. Regarding T4, both the typical and atypical BD groups had significantly higher values than the simple anxiety group. It has been suggested that increased T4 after hospitalization is positively correlated with the severity of BD and that its rate of decline is associated with a better prognosis [43]. These correlations show that T4 can stand alone as an endocrine indicator that distinguishes between BD and anxiety disorders. Similarly, regarding FT4, both the typical and atypical BD groups had significantly higher values than the simple anxiety group. Southwick et al. [44] also observed temporarily increased FT4 in hospitalized BD patients during both manic and depressive episodes. Regarding ACTH and PTC, the typical BD group had significantly lower values than the other two groups, indicating that although the typical and atypical BD groups had the same trend towards HPA axis impairment, the patients in the atypical group retained some characteristics in common with anxiety disorders. Furthermore, many studies have found that both generalized anxiety disorder and obsessive-compulsive disorder patients had increased levels of ACTH and PTC [45–47], making it difficult to distinguish atypical BD from anxiety disorders based on these factors.

### Differences in the inflammatory cytokine index among the three groups

The results of our study suggest that the rates of abnormality for the inflammatory cytokines did not differ significantly between the typical BD group and the atypical BD group. Furthermore, the rates of abnormal IL-6, IL-8, TNF-α and CRP in those two groups were all significantly higher than those of the simple anxiety group, providing additional confirmation that atypical BD does exist. There are some data showing that increased immune and inflammatory signals may be closely related to the pathological aetiology of mood disorders and, therefore, may become new therapeutic targets for the development of more-effective treatments [48].

The pairwise comparison results show that the IL-6, TNF-α and CRP values of the atypical BD group were significantly higher than those of the other two groups, while the IL-10 value was significantly lower than those of the other two groups. It is well known that IL-6 and TNF-α are pro-inflammatory cytokines, while IL-10 is an anti-inflammatory cytokine. Our results suggest that patients in the atypical BD group may have more-serious inflammatory and immune responses and that their prominent changes in inflammatory cytokines could be another major feature distinguishing them from patients with ordinary anxiety disorders. In recent years, an increasing number of studies have found an imbalance in pro-inflammatory and anti-inflammatory cytokines in BD patients. For instance, the level of TNF-α in BD patients increased during both depressive and manic episodes [49]. Kauer-Sant’Anna et al. [50] also reported that the levels of TNF-α and IL-6 increased in BD patients during their early episodes. Other studies have confirmed that pro-inflammatory cytokines, such as IL-6 and TNF-α, can stimulate the hepatic biosynthesis system to produce CRP [51]. As a result, the level of CRP in our atypical BD group increased, as did the levels of TNF-α and IL-6. However, we were surprised that we failed to find significant differences between the typical BD group and the simple anxiety group in terms of inflammatory cytokine indicators. A possible reason for this result is that the first onset of the patients enrolled in the typical BD group was anxiety disorders in all cases and that they had been diagnosed with BD. Therefore, they could not avoid the use of mood stabilizers for treatment, and related research has confirmed that mood stabilizers can lower the conventional starting components of the immune inflammatory signalling pathways [48]. It is easy to understand why the characteristics of the typical BD patients differed from those of the atypical BD patients but were similar to those of the anxiety patients.

### The rate of progression to mania

The rate of progression to mania illustrates that throughout the process, some of the anxiety patients appeared to manifest manic symptoms quickly, which further demonstrates that a transition from anxiety to BD does exist. However, we removed them from the last analysis because these patients might possess their own features different from those of atypical BD, allowing them to transform quickly. In other words, a patient with atypical BD may never develop typical BD and may only sustain the atypical symptoms as a variation of BD, or they may eventually develop BD over a long period of time. This proposition needs to be tested in further research.

## Conclusions

As a result of our analyses and comparisons, we conclude that the progression from anxiety disorders to bipolar disorder may represent a continuous disease process, with some specific anxiety disorders (namely, atypical BD) representing the phase of abnormal rhythm within the BD disease spectrum. Only patients who presented inherent characteristics similar to those of BD, that is, those in the prodromal period, can continue to develop a pathological rhythm and ultimately present with BD. However, these special BD patients also have some unique features that could represent the turning points at which anxiety disorders develop into BD; these features include severe suicidal ideation, changes from HPA axis to HPT axis activation, and elevated inflammatory cytokines. Finally, our study presents a methodology that can be used to explore other psychiatric diseases that also might have prodrome, onset and complication periods from the perspective of biorhythms.

## Additional file

Additional file 1: Demographic data and relevant scales. (DOCX 22 kb)

## Abbreviations

ACTH: Adrenocorticotropic Hormone
AD: Anxiety disorder
BD: Bipolar disorder
CGI: Clinical Global Impression
CRP: C- Reactive Protein
FT3: Free Triiodothyronine
FT4: Free Thyroxine
HAMA: Hamilton Anxiety Scale
HPA axis: Hypothalamic - Pituitary - Adrenal axis
HPT axis: Hypothalamic - Pituitary - Thyroid axis
ICD-10: International Classification of Diseases, Tenth Edition
IL-10: Interleukin-10
IL-6: Interleukin-6
IL-8: Interleukin-8
NEO-FFI: NEO Five-factor Inventory
NESARC: National Epidemiological Survey on Alcohol and related diseases condition
NIMH: National Institute of Mental Health
OCD: Obsessive-compulsive disorder
PTC: Cortisol
T3: Triiodothyronine
T4: Thyroxine
TNF-α: Tumor Necrosis Factor -α
TSH: Thyroid Stimulating Hormone
YBOCS: Yale - Brown Obsessive Compulsive Scale
YMRS: Young Mania Rating Scale
