# Supplementary material for: Do some anxiety disorders belong to the prodrome of bipolar disorder? A clinical study combining retrospective and prospective methods to analyse the relationship between anxiety disorder and bipolar disorder from the perspective of biorhythms
Source: BMC Psychiatry. 2017 Oct 24;17:351. doi: 10.1186/s12888-017-1509-6 (PMC5655950; doi:10.1186/s12888-017-1509-6)
Supplement: Additional file 1: — Demographic data and relevant scales. (DOCX 22 kb) [file 12888_2017_1509_MOESM1_ESM.docx]

**The basic demographic questionnaire**

Note: please choose the corresponding number according to your actual condition. Just draw ”√” below the number you choose and write the actual information on the” “where needed.

**The demographic data:**

Birth date Age (years)

Sex：1.male 2.female

Nationality：1.Han 2.Minority, please note

Birth place：1.Sichuan 2.Chongqing 3.Guizhou 4. others, please note

Residence：1.City 2.Village

Education：1.Primary 2. Middle 3.High 4. Bachelor 5.Master

Marriage：1.Unmarried 2.Married 3.Divorced 4.Remarried 5.Widowed

Work：1. None 2. Student 3.Worker 4.Teacher 5.Doctor 6.Civil servant 7.Staff 8.Others, please note

Family history of psychotics：1. No 2.Yes, please note

**The clinical features of disease：**

**If you are not sure about your type, please consult your doctor**

Type of bipolar disorder (BD): 1.Hypomania 2. Non psychotic mania

(Only the patients diagnosed with BD fill in this item)

3. Psychotic mania 4. Mixed state 5.Remission 6. Mild depression 7. Non psychotic major depression 8.Psychotic major depression

Type of anxiety: 1. generalized anxiety disorder (GAD)

(Only the patients diagnosed with anxiety fill in this item)

2. obsessive-compulsive disorder (OCD)

3. Stress related disorders (including acute stress disorder, and post-traumatic stress disorder)

4. Panic Disorder

Is it your first onset? 1. Yes 2.No, if no, what’s the number of your onsets? and what’s the type of your first onset?

1. GAD

2. OCD

3. Stress related disorders

4. Panic Disorder

The age of your first onset:

The duration of months since you have been diagnosed with BD:

(Only the patients diagnosed with BD fill in this item)

The duration of months since you have been diagnosed with anxiety:

(Only the patients diagnosed with anxiety fill in this item)

Frequency of onset (number/year):

Ever had a drug abuse: 1. No 2. Yes

If have other diseases (such as diabetes, hypertension,and so on):

1.No 2. Yes, please note

**Please evaluate the severity of your suicidal ideation:**

0. None

1. Between 0 and 2

2. Bored with life, have a fleeting moment of suicidal thoughts

3. Between 2 and 4

4. Often with suicidal ideation, and think that suicide is a possible self-solving method, but there is no real plan for suicide

5. Between 4 and 6

6. A suicidal plan has been set and you are waiting for the opportunity to make it true

**The Hamilton Anxiety Scale (HAMA)**

Reference: Sun XL. Psychiatry. 3rd ed. Beijing:Higher Education Press; 2008.

**The Young Mania Rating Scale (YMRS)**

Reference: Sun XL. Psychiatry. 3rd ed. Beijing:Higher Education Press; 2008.

**The Yale-Brown Obsessive-Compulsive Scale (YBOCS)**

Reference: Sun XL. Psychiatry. 3rd ed. Beijing:Higher Education Press; 2008.

**The Clinical Global Impression scale (CGI)**

Reference: Sun XL. Psychiatry. 3rd ed. Beijing:Higher Education Press; 2008.

**The NEO Five-Factor Inventory (NEO-FFI)**

Reference: Yao RS, Liang LY. Analysis of the application of simplified NEO-FFI to undergraduates. Chin J Clin Psychol, 2010; 18(4): 457-9.
